# Association of Motoric Cognitive Risk Syndrome with Sarcopenia and Systemic Inflammation in Pre-Frail Older Adults

**DOI:** 10.3390/brainsci13060936

**Published:** 2023-06-09

**Authors:** Reshma Aziz Merchant, Yiong Huak Chan, Denishkrshna Anbarasan, Ivan Aprahamian

**Affiliations:** 1 Division of Geriatric Medicine, Department of Medicine, National University Hospital, 1E Kent Ridge Road, Singapore 119228, Singapore; 2 Department of Medicine, Yong Loo Lin School of Medicine, National University of Singapore, Singapore 119228, Singapore; 3 Biostatistics Unit, Yong Loo Lin School of Medicine, National University of Singapore, Singapore 119077, Singapore; medcyh@nus.edu.sg; 4 Geriatrics Division, Department of Internal Medicine, Jundiai Medical School, Jundiai 13202-550, SP, Brazil

**Keywords:** tumor necrosis factor-α, interleukin-10, body fat percentage

## Abstract

Motoric cognitive risk syndrome (MCR) is defined by the presence of slow gait and subjective cognitive decline. It is well recognized as a prodrome for dementia, but the biological mechanism and trajectory for MCR are still lacking. The objective of this study was to explore the association of MCR with body composition, including sarcopenia and systemic inflammation, in pre-frail older adults in a cross-sectional study of 397 pre-frail community-dwelling older adults. Data on demographics, physical function, frailty, cognition (Montreal Cognitive Assessment (MoCA)), perceived health and depression were collected. Body composition was measured using a bioelectrical impedance analyzer. Systemic inflammatory biomarkers, such as progranulin, growth differentiation factor-15 (GDF-15), interleukin-10 (IL-10), interleukin-6 and tumor necrosis factor-α (TNF-α), were collected. Univariate and multivariate logistic regression were used to analyze the association between MCR, body composition, sarcopenia and systemic inflammatory biomarkers. The prevalence of MCR was 14.9%. They were significantly older and there were more females, depression, functional impairment, lower education, physical activity and MoCA scores. Body fat percentage (BF%), fat mass index, fat to fat free mass ratio (FM/FFM) and sarcopenia prevalence were significantly higher in MCR. Serum GDF-15 and TNF-α levels were highest with progranulin/TNF-α and IL-10/TNF-α ratio lowest in MCR. Compared to healthy patients, MCR was significantly associated with sarcopenia (aOR 2.62; 95% CI 1.46–3.17), BF% (aOR 1.06; 95% CI 1.01–1.12), FMI (aOR 1.16; 95% CI 1.02–1.30) and FM/FFM (aOR 6.38; 95% CI 1.20–33.98). The association of IL-10 to TNF-α ratio (aOR 0.98, 95% CI 0.97–0.99) and IL-10 (aOR 2.22, 95% CI 0.05–0.98) with MCR were independent of sarcopenia and BF%. Longitudinal population studies are needed to understand the role of body fat indices and IL-10 in pre-frail older adults with MCR and trajectory to dementia.

## 1. Introduction

With a rapidly aging population worldwide, countries will experience shifting disease burden with rises in non-communicable diseases and common conditions in old age, such as dementia, sarcopenia and frailty, putting a strain on health and long-term care expenditure and increased caregiver burden. Dementia and frailty are both risk factors for disability, often co-exist in the last few years of life and share similar trajectories [1]. Motoric cognitive risk syndrome (MCR), a predementia syndrome also known as cognitive frailty, is defined by the presence of slow gait and subjective cognitive decline. The concept of MCR was initially described by Verghese and colleagues in 2013 [2]. Depending on the population studied, the prevalence of MCR varies between 6.3% and 18.0% [3,4,5,6,7,8,9,10,11]. MCR is significantly associated with an increased risk of dementia, as shown initially in the Einstein Aging Study population and validated in other countries where MCR participants had more than threefold increased risk of dementia and twelvefold increased risk of vascular dementia over a median follow up of 36.9 months [12,13]. Both slow gait and lower scores in executive domains have been shown to be predictive of dementia [14]. MCR is also associated with high BMI, frailty and pre-frailty, depression, pain, falls, fractures, disability and increased mortality [4,9,15,16]. While treatment for dementia is still in evolution, MCR has been shown to be reversible with physical exercise, cognitive and social activities, nutrition and symptomatic treatment, such as antidepressants and counselling [17,18].

Frailty is a dynamic multidimensional syndrome characterized by loss of physiological reserve predisposing a person to adverse outcomes from stressors [19]. Similar to MCR and dementia, frailty is associated with falls, increased morbidity and mortality [20]. Pre-frailty lies along the continuum of frailty and is a known risk factor for the development of frailty, affecting approximately 40% of older adults [19,21,22]. Frailty and cognition are closely related, where mild cognitive impairment (MCI) and dementia can accelerate frailty, and frailty accelerates cognitive decline in dementia, independent of underlying pathological burden [23]. Shen et al. showed that MCR and slow gait, but not subjective cognitive decline, are independently associated with frailty [20]. Decreased lean mass and high body fat percentage have been shown to be associated with increased dementia and frailty risk [24,25,26].

Systemic inflammation, which is prevalent in frailty, obesity and aging, is associated with cognitive impairment, slow gait and mobility disability [27]. Chronic inflammation is a known cause for neuronal loss, and many pro-inflammatory cytokines, such as tumor necrosis factor alpha (TNF-α) and interleukin-6 (IL-6), are found to be elevated in Alzheimer’s dementia (AD) [28]. Interleukin-10 (IL-10) is an immunosuppressive cytokine and plays an important role in modulating inflammation and inhibiting the production of pro-inflammatory cytokines [29]. IL-10 is implicated in various neurological conditions, such as multiple sclerosis, Parkinsons’s disease and AD [29]. Other cytokines, such as growth-differentiated factor-15 (GDF-15) and progranulin, play important roles in neurogenesis and cognition as well as inhibiting the action of pro-inflammatory cytokines [30,31]. Reduced progranulin levels have been associated with neuroinflammation, abnormal microglial activation, neuronal loss and increased risk for developing dementia [30]. GDF-15 is generated in the choroid plexus, damaged neurons and microglial cells and found to be elevated in persons with neurodegenerative disease [31]. There are limited studies on body composition changes in pre-frail older adults with MCR and systemic inflammatory biomarkers. We hypothesize that MCR individuals might present with higher body fat indices, low lean mass or sarcopenia and increased pro-inflammatory systemic biomarkers. This study aims to explore the association of MCR, slow gait and subjective cognitive decline with body composition, including sarcopenia and systemic inflammation, in pre-frail older adults.

## 2. Methods

### 2.1. Study Participants and Design

**Figure 1:**
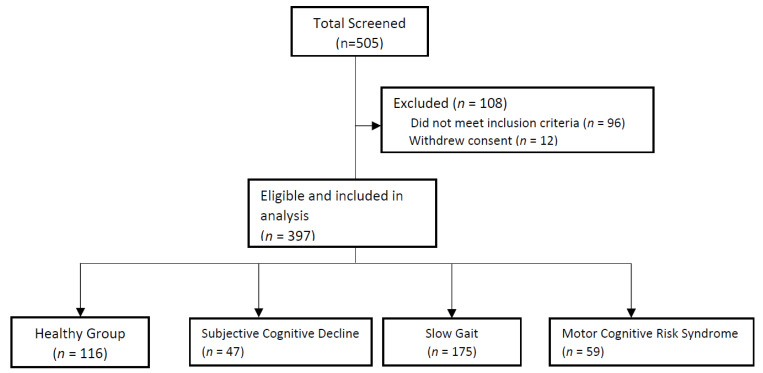
Participant screening and group allocation.

This is a cross-sectional analysis of baseline data for participants initially recruited for multidomain intervention study in pre-frail older adults ≥60 years old from the community and primary care centers (Figure 1). Recruited participants should be able to provide consent and follow instructions. Exclusion criteria included nursing home residents, dementia, presence of pacemaker or defibrillator and underlying psychiatric conditions. This study conformed to the principles of the Declaration of Helsinki and was approved by The National Healthcare Group Domain Specific Review Board (Reference: 2018/01183 and 2019/00017). Informed consent was obtained from all participants involved in the study.

Gait speed was measured over 4 m, and slow gait was defined as <1 m/s [32]. Subjective cognitive decline was defined based on a question from the 15-item Geriatric Depression Scale as “do you feel you have more problems with memory than most?” [33]. MCR was defined by the presence of both subjective cognitive decline and slow gait. Pre-frail was defined based on the FRAIL (Fatigue, Resistance, Aerobic, Illness and Loss of Weight) questionnaire score of 1–2 out of 5 components [19]. Participants were categorized into four groups: (i) healthy (no subjective cognitive decline or slow gait), (ii) subjective cognitive decline (subjective cognitive decline without slow gait), (iii) slow gait (slow gait without subjective cognitive decline) and (iv) MCR.

### 2.2. Co-Variates

Trained research assistants administered the study protocol, gathering information on demographics, medications, chronic diseases, cognition, falls, sarcopenia, depression, functional status, pain, nutrition and perceived health. Polypharmacy was defined as taking ≥5 medications daily and multimorbidity as ≥2 chronic diseases. EuroQoL-5D was used to evaluate pain and perceived health using the EuroQoL Visual Analogue Scale [34]. Activities of daily living (ADL) were evaluated using Katz’s ADL questionnaire and instrumental activities of daily living (IADL) using the Lawton and Brody’s IADL questionnaire [35,36]. Cognition was assessed using the Montreal Cognitive Assessment (MoCA) [37] and depression using the 15-item Geriatric Depression Scale when participants scored ≥5 points [33]. Nutrition was screened using the Mini Nutritional Assessment short form [38]. The Rapid Physical Assessment (RAPA) was used to assess physical activity [39].

Maximum handgrip strength (HGS) was measured in a seated position using the Jamar hand dynamometer on with elbow flexed at 90°. Low HGS was defined as <28 kg for males and <18 kg for females [32]. The short physical performance battery test (SPPB) and its 3 components, namely balance, gait and chair stand, were measured with gait speed measured at 4 m, with 3 m of acceleration and deceleration path. Waist circumference was measured between the last rib and the iliac crest.

### 2.3. Body Composition

Body composition was assessed using the InBody S10 multi-frequency bioelectrical impedance analyzer. Readings for body fat percentage, fat mass (FM), fat free mass (FFM), appendicular skeletal muscle (ASM) and visceral fat area (VFA) were obtained. Fat Mass Index (FMI), Fat Free Mass Index (FFMI) and Appendicular Skeletal Muscle Index (ASMI) were obtained by dividing FM, FFM and ASM by height squared, respectively. Diagnosis of sarcopenia was based on the 2019 Asian Workgroup for Sarcopenia (AWGS) criteria [32].

### 2.4. Inflammatory Biomarkers

The TNF-α, IL-6, GDF-15, IL-10 and progranulin cytokines were measured by accredited hospital-based laboratory. Immunoenzymetric assay was used to measure TNF-α cytokine, with a detection range between 1.0 and 498 pg./mL. IL-6 was measured using the electrochemiluminescence immunoassay (ECLIA), with a detection range between 1.5 and 50,000 pg./mL. Enzyme-linked immunosorbent Assay was used to measure IL-10, with a detection range of 2.0–400.0 pg./mL and GDF-15 with a detection range of 2.0–2400 pg./mL. Progranulin was measured using sandwich enzyme immunoassay with a detection range of 0.17–400 ng/mL. The ratio of progranulin to TNF and IL-10 to TNF-α was also calculated.

### 2.5. Statistical Analysis

All analyses were performed using SPSS 28.0 with statistical significance set at 2-sided 5%. Descriptive analyses for categorical and continuous variables were presented as frequencies with percentages and mean ± standard deviation, respectively. Univariate analysis for numerical measures across the groups was performed with Welch test to account for the unequal sample sizes and Games–Howell post hoc for pairwise comparisons. Chi Square test with Bonferroni correction was used for categorical variables.

Multinomial regression was performed to explore the association of subjective cognitive decline, slow gait and MCR participants with sarcopenia, body composition and systemic inflammatory biomarkers, when compared to the healthy participants. Multiple adjustments were made for age, gender, ethnicity, education years, chronic diseases, polypharmacy, nutritional status and physical activity. Unadjusted and adjusted odds ratios with 95% confidence interval were reported.

## 3. Results

### 3.1. Characteristics of the Participants

Recruitment and screening of participants are reflected in Figure 1, while the characteristics of participants were categorized into healthy, subjective cognitive decline, slow gait and MCR, and they are shown in Table 1. Of the 397 pre-frail older adults, mean age was 72.5 ± 5.8 years, 61.9% female, education level 7.9 ± 4.4 years and 86.2% were of Chinese ethnicity. MCR was present in 59 (14.9%), slow gait in 175 (44.1%) and subjective cognitive decline in 47 (11.8%). Participants with MCR (74.4 ± 6.4 years) and slow gait (73.9 ± 5.5 years) were significantly older compared with healthy and subjective cognitive decline (70.2 ± 4.9 years and 71.6 ± 5.5 years, respectively). Slightly more than three-quarters of the MCR participants were females compared with about half in the other groups. Both the MCR and slow gait group had higher body mass index (BMI) compared to the subjective cognitive decline and healthy group (25.95 ± 5.2 kg/m^2^ and 26.02 ± 4.9 kg/m^2^ versus 24.65 ± 3.8 kg/m^2^ and 24.58 ± 4.1 kg/m^2^, respectively), significantly lower education level (6.6 ± 4.0 years and 7.1 ± 4.1 years versus 8.3 ± 5.2 years and 9.4 ± 4.1 years, respectively) and lower MoCA scores (23.5 ± 4.9 and 24.8 ± 4.1 versus 26.0 ± 4.0 and 27.2 ± 2.6, respectively).

There was a higher prevalence of depression amongst the MCR group (52.5%) compared with slow gait (25.1%), subjective cognitive decline (29.8%) and healthy (17.2%) groups. In addition, the majority of MCR participants had at least one ADL or IADL impairment (33.9% and 42.4%, respectively). The MCR group compared with the healthy group had the lowest physical activity level (2.8 ± 1.5 vs. 3.5 ± 1.5). The MCR and slow gait group compared with subjective cognitive decline and healthy group had slower maximum gait speed (0.8 ± 0.2 m/s and 0.8 ± 0.2 m/s versus 1.2 ± 0.2 m/s and 1.2 ± 0.2 m/s respectively) and lower SPPB total scores (8.4 ± 2.3 and 9.0 ± 2.2 versus 10.7 ± 1.5 and 11.0 ± 1.3, respectively). Furthermore, MCR participants had the lowest maximum handgrip strength, and two-thirds had low handgrip strength.

### 3.2. Motoric Cognitive Risk Syndrome, Subjective Cognitive Decline, Slow Gait, Sarcopenia and Body Composition Associations

Fat mass indices, reflected by body fat percentage, FMI and FM to FFM ratio, were significantly higher in the MCR compared with the healthy group (body fat %: 36.2% + 9.3 versus 31.1% + 8.8; FMI: 9.7 + 4.1 versus 7.9 + 3.2; FM/FFM: 0.6 + 0.2 versus 0.5 + 0.2, respectively). However, no significant differences in ASMI and VFA were observed. Sarcopenia according to the AWGS definition was more prevalent in the MCR group (28.8%), followed by slow gait (20.6%) and lowest amongst the subjective cognitive decline (2.1%) and healthy group (0.9%).

Table 2 summarizes the results of unadjusted and adjusted analysis of the association between the subjective cognitive decline, slow gait and MCR participants, with waist circumference, body composition and sarcopenia, compared to the healthy group. The results were adjusted for age, gender, ethnicity, education years, chronic diseases, polypharmacy, nutritional status and physical activity. MCR was significantly associated with sarcopenia (aOR 2.62; 95% CI 1.46–3.17), BF% (aOR 1.06; 95% CI 1.01–1.12), FMI (aOR 1.16; 95% CI 1.02–1.30) and FM to FFM ratio (aOR 2.56; 95% CI 1.62–3.33). Slow gait was significantly associated with sarcopenia (aOR 1.90; 95% CI 1.14–2.40) and increased waist circumference (aOR 1.04; 95% CI 1.01–1.07).

### 3.3. Motoric Cognitive Risk Syndrome, Subjective Cognitive Decline, Slow Gait and Systemic Inflammatory Biomarkers

Of the 107 participants with complete serum biomarker data (Table 3), participants with MCR and slow gait compared with subjective cognitive decline and healthy groups had higher levels of GDF-15 (1132.4 ± 602.5 pg./mL and 1100.47 ± 565.0 pg./mL versus 981.8 ± 506.4 pg./mL and 715.6 ± 315.4 pg./mL, respectively). MCR participants had the highest TNF-α level (9.5 ± 2.6 pg./mL) compared with slow gait (8.5 ± 2.8 pg./mL), subjective cognitive decline (7.1 ± 1.4 pg./mL) and healthy (7.0 ± 1.7 pg./mL) groups and, conversely, the lowest progranulin/TNF-α and IL-10/TNF-α ratio. Table 4 outlines the results of unadjusted and adjusted analysis of the association between the groups with systemic inflammatory biomarkers, compared to the healthy group. Model 2 was adjusted for age, gender, ethnicity, education years, chronic diseases, polypharmacy, nutritional status, physical activity and sarcopenia, and Model 3 included Model 2 together with body fat percentage. In Model 2, MCR was associated with increased TNF-α (aOR 1.23; 95% CI 1.04–1.46), reduced IL-10 to TNF-α ratio (aOR 0.98, 95% CI 0.97–0.99) and reduced PRGN/TNF- α ratio (aOR 0.98; 95% CI 0.92–0.99). In Model 3, after adjustment for body fat percentage, MCR was significantly associated with reduced IL-10 to TNF-α ratio (aOR 0.98, 95% CI 0.97–0.99) and reduced IL-10 (aOR 0.22; 95% CI 0.05–0.98).

## 4. Discussion

MCR, slow gait and subjective cognitive decline are considered as harbingers of dementia, and studies have shown that MCR carries the highest burden for poor functional outcomes, depression, pain, falls, institutionalization and mortality [2,4,15,16,27]. A better characterization of MCR individuals will allow for early identification and the potential development of preventive interventions against the development of dementia. In our study, pre-frail participants with MCR showed a significant association with sarcopenia, body fat percentage and FM to FFM ratio, which was not evident in the other groups. MCR was also significantly associated with low IL-10 and IL-10 to TNF-α ratio after adjusting for sarcopenia and body fat percentage. Aging is associated with impaired neuromuscular junction function, ectopic fat deposition and low-grade inflammation, causing mitochondrial dysfunction, insulin resistance, dysfunctional adipokine and myokine release, causing sarcopenia and cognitive impairment [24,25,26,40,41].

Neurogenesis and cognitive function are mediated through muscle–brain communication, which, in turn, is dependent on the endocrine capacity of skeletal muscle [42]. Sarcopenia is an age-related loss of muscle strength, quality, quantity or reduced physical performance, which is independently associated with negative outcomes, such as falls, fracture and mortality [43,44]. The prevalence of sarcopenia in our study participants with MCR was 28.8%, which was much higher than our healthy group of 0.9%. There is a huge variation in the population prevalence of sarcopenia of 10% to 27% due to heterogeneity in the population studied and the screening tools used [45]. In our study, MCR was also significantly associated with increased FM to FFM ratio. Both FM and FFM have differing physiological roles, where the sarcopenia, cardiometabolic and disability risk have been shown to be dependent on the relative contribution of each of the components [46,47]. Total body fat and fat infiltration in the muscle, which is also known as myosteatosis, can affect muscle contractility, muscle mass, reduced release of myokines such as irisin and increased release of pro-inflammatory myokines and adipokines [44,48]. Irisin has been associated with increased myogenesis and neurogenesis, which has been shown to be mediated through the release of brain-derived neurotropic factor [49]. MCR has been associated with declines in global cognitive function, attention, processing speed and executive function [50]. Tesser et al. showed that older adults with low muscle mass experienced greater cognitive decline, specifically in the executive domain over 3 years [51]. Data from the study on Global Ageing and Adult Health (SAGE) in Mexico showed that the prevalence of mild cognitive impairment increased at a rate of 0.8% annually in non-sarcopenic and nearly 1.5% in sarcopenic people [52]. Studies have shown that sarcopenia may be reversible through resistance exercise and a high-protein diet [53].

Very little is known on how the interaction between the basic biology of aging, chronic inflammation and cellular senescence affect body composition changes and inflammation in MCR. Gait speed is one of the diagnostic criteria for MCR and sarcopenia [12,43]. Results from the Korean Frailty and Aging Cohort Study showed that gait speed may be a mediator between sarcopenia and cognitive impairment [54]. A systematic review has shown that the impact of gait speed alone on future risk of dementia is, at most, modest [13]. Declines in both gait speed and memory have greater prognostic value in predicting dementia risk, as both share a common neural pathway and are indicative of neurodegenerative disease rather than musculoskeletal problems [13]. MCR is known to be associated with low grey matter volume in the prefrontal and premotor cortex, which could affect gait speed [55]. Handgrip strength, which is a measure of muscle strength and core determinant of sarcopenia, has been shown to predict 10-year cognitive decline [56]. Higher handgrip strength has been associated with larger hippocampal volume and transition to robust in the group with physio-cognitive decline syndrome [57,58]. Exercises which showed improvement in gait speed and handgrip strength also showed improvement in attention and cognition in community-dwelling older adults with MCR, further strengthening the intimate relationship between cognitive and physical function [18,59].

TNF-α was significantly elevated in MCR and slow gait, but significant association was only seen in MCR participants, independent of sarcopenia but not obesity, defined by body fat percentage. Only low IL-10 and IL-10 to TNF-α ratio were significantly associated with MCR, independent of sarcopenia and body fat percentage. IL-10 is a well-recognized immunomodulator and immunosuppressive cytokine secreted by almost all leukocytes, keratinocytes and epithelial cells responsible for the resolution of immune response and tissue repair. It inhibits the release of various pro-inflammatory cytokines, such as TNF- α and IL-6. In the nervous system, glial cells, especially microglia (resident macrophages), astrocytes and oligodendrocytes, are responsible for maintaining neural homeostasis and preventing neuronal death from chronic neuroinflammation through IL-10 expression. The stimulation for release in the central nervous system is complex and poorly understood. Infections or tissue injury activate microglia to the M1 phenotype, which is pro-inflammatory, followed by M2 phenotype activation, which is anti-inflammatory [60]. It is not known if imbalance between M1 and M2 microglial activation contributes to neurotoxicity. Studies show that an increase in IL-10 may be transient, whereas TNF-α may be elevated chronically, and there is a possible role of low IL-10 to TNF-α ratio, contributing to severe knee osteoarthritis [61]. Low IL-10 to TNF-α ratio was significantly associated with MCR in our pre-frail study participants. Like our MCR participants, AD patients have been shown to have weak expression of IL-10, and depressed levels of IL-10 have also been seen in pain and clinical symptoms in Parkinson’s disease. Early animal studies show that IL-10 pre-treatment in mice protects ventral mesencephalic neurons against the toxic effects of lipopolysaccharide through upregulation of brain-derived neurotrophic factor and inhibition of TNF-α release [29].

Progranulin is also recognized as an adipokine and, together with TNF-α and GDF-15, is known to be associated with obesity. TNF-α acts through the TNF receptor 1 (pro-inflammatory) and TNF receptor 2 (anti-inflammatory) [62]. In the AD brain, TNF-α has greater affinity for TNFR1 supported further by postmortem studies where TNFR1 levels were higher and TNFR2 levels lower in AD brains [62]. TNF-α inhibitors have been shown to be associated with a lower risk of dementia in patients with rheumatoid arthritis and animal studies, but there are concerns of adverse events and the blocking physiological role of TNF-α through TNFR2 [63,64]. Systemic inflammation is prevalent in obesity, and it is one of the key determinants of failure of anti-TNF agents, with each unit increase in BMI associated with 6.5% of failing therapy [65].

GDF-15, also known as macrophage inhibitory cytokine-1, is expressed in most organs, including damaged neurons, and it is a marker of mitochondrial dysfunction and elevated in myopathies and neurodegenerative disease [66]. It has both a protective effect, where it plays a role in modulating immune function and improving insulin sensitivity, as well as a negative systemic effect, where it serves as a prognostic biomarker for cancer and cardiovascular disease [31,66]. GDF-15 is known to be associated with slow gait and functional impairment [31]. GDF-15 was significantly elevated in MCR and slow gait participations but not associated in the final model, possibly due to other interacting factors or small sample size. Progranulin is expressed in epithelial cells, neurons, chondrocytes and immune cells [67]. It has diverse action from tissue repair, wound and bone healing, neurogenesis and anti-inflammation mediated through TNFR2 to tumorigenesis where it is elevated in many cancers [67]. Progranulin blocks the TNF-α signaling pathway, and increased progranulin to TNF-α ratio may have a role to play in neutralizing the pro-inflammatory effect of TNF-α [68]. While there was no significant difference in progranulin levels between the groups, reduced progranulin to TNF-α level was significantly associated with MCR, independent of sarcopenia but not when adjusted for body fat percentage.

Almost three-quarters of the participants with MCR were women in our study. A possible explanation includes the higher incidence of frailty and prefrailty in women, as shown in a recent systematic review [69]. The prevalence of depression in our MCR participants was double that of the slow gait participants. The association between depression and MCR has been shown in many prior studies [16]. The mechanism of depression in MCR is not known but could be mediated by cytokines, such as TNF-α, fat mass, grey matter volume and handgrip strength [58,70].

### 4.1. Strengths and Limitations

The strength of our study includes the involvement of community-dwelling pre-frail older adults, robust co-variates and objective measurements of body composition and physical performance. The limitation is the cross-sectional design in pre-frail older adults, which does not allow us to determine the longitudinal changes in gait speed, body composition and cognition. Due to this, we are unable to deduce if sarcopenia or fat indices could be an early signature in pre-frail participants at risk of MCR or a consequence of MCR. Second, the majority of the study participants were pre-frail and from the Chinese ethnic group, where our previous study highlighted overreporting of subjective cognitive decline in this ethnic group; hence, the results from this study cannot be generalized to other ethnic groups or populations [9]. Third, chronic diseases, subjective cognitive decline and falls were self-reported and may be subject to recall bias, especially in participants with MCR. However, participants with subjective cognitive decline and MCR did have lower cognitive scores and reported an increased number of falls, which is in keeping with other published studies. Fourth, the subgroup sample sizes were unequal, but the findings were adjusted using the Welch test. Fifth, body composition measured via BIA may be affected by edema, hydration and fasting. Lastly, as this is a cross-sectional study, causal inferences cannot be assumed.

### 4.2. Study Highlights and Future Directions

Our study is one of the first to show an association of IL-10 with MCR in pre-frail older adults, which requires further validation at the population level and could serve as a future therapeutic target. In addition, our study showed a significant association of MCR with sarcopenia, as well as fat mass indices in pre-frail older adults, with a significant increase in TNF-α and GDF-15. It is not known if interventions to reduce body fat in this group will reverse or slow down the progress of MCR and reduce chronic inflammation. The role of individual systemic inflammatory biomarkers in mediating the negative outcomes of MCR, such as a decline in physical performance and depression, needs to be validated in longitudinal studies. Future studies are needed to validate the above findings at a larger population level, augmented by biomarkers and functional imaging. The concept of body fat, inflammation and balance between IL-10 and TNF-α, cellular senescence, mitochondrial dysfunction and sarcopenia needs to be further elucidated in the MCR participants and the role as a mediator for progression to dementia.

## 5. Conclusions

MCR was significantly associated with IL-10, IL-10 to TNF-α ratio, sarcopenia, body fat indices and systemic inflammation. Future longitudinal population studies are needed to understand the pathogenesis of MCR, the trajectory of progression to dementia and the role of systemic inflammation and sarcopenia in predicting MCR onset and dementia in pre-frail older adults.

## Figures and Tables

**Table 1:** Baseline characteristics.

	Healthy	Subjective Cognitive Decline	Slow Gait	Motoric Cognitive Risk Syndrome	*p* Value
	*n* = 116 (29.2%)	*n* = 47 (11.8%)	*n* = 175 (44.1%)	*n* = 59 (14.9%)	
**Demographics**					
Age, years	70.2 ± 4.9 ^a^	71.6 ± 5.5	73.9 ± 5.6	74.4 ± 6.4 ^a^	<0.001
Gender					0.033
Male	50 (43.1)	19 (40.4)	79 (45.1)	14 (23.7)	
Female	66 (56.9)	28 (59.6)	96 (54.9)	45 (76.3)	
Ethnicity					0.047
Chinese	109 (94.0)	41 (87.2)	138 (78.9)	50 (84.7)	
Malay	2 (1.7)	2 (4.3)	18 (10.3)	3 (5.1)	
Indian	5 (4.3)	4 (8.5)	16 (9.1)	6 (10.2)	
Others	0 (0.0)	0 (0.0)	3 (1.7)	0 (0.0)	
BMI, kg/m^2^	24.6 ± 4.1 ^a,b^	24.7 ± 3.8 ^c,d^	26.0 ± 4.9 ^a,c^	26.0 ± 5.2 ^b,d^	0.031
Waist circumference (cm) *	89.0 ± 12.7	88.3 ± 10.0	94.5 ± 13.4	92.5 ± 13.6	0.006
Education, years	9.4 ± 4.1 ^a^	8.3 ± 5.2	7.1 ± 4.1	6.6 ± 4.0 ^a^	<0.001
Chronic Disease					
Hypertension	77 (66.4)	29 (61.7)	122 (69.7)	41 (69.5)	0.692
Hyperlipidemia	86 (74.1)	34 (72.3)	136 (77.7)	44 (74.6)	0.869
Diabetes	45 (38.8)	19 (40.4)	91 (52.0)	26 (44.1)	0.138
Stroke	8 (6.9)	3 (6.4)	17 (9.7)	4 (6.8)	0.767
Multi-morbidity	86 (74.1)	32 (68.1)	144 (82.3)	44 (74.6)	0.132
Polypharmacy	24 (20.7)	12 (25.5)	59 (33.7)	16 (27.1)	0.100
Perceived Health Rating	71.8 ± 14.3 ^a^	71.5 ± 11.3	68.7 ± 14.4	64.8 ± 15.3 ^a^	0.012
RAPA Total	3.5 ± 1.5 ^a^	3.2 ± 1.6	3.3 ± 1.6	2.8 ± 1.5 ^a^	0.031
Rare/Light Activity	64 (55.2)	31 (66.0)	109 (62.3)	44 (74.6)	0.086
Moderate/Vigorous Activity	52 (44.8)	16 (34.0)	66 (37.7)	15 (25.4)	
MoCA, total	27.2 ± 2.6 ^a,b^	26.0 ± 4.0 ^c,d^	24.8 ± 4.1 ^a,c^	23.5 ± 4.9 ^b,d^	<0.001
≥1 Fall in 12 months	27 (23.3)	9 (19.1)	36 (20.6)	22 (37.3)	0.085
Depression	20 (17.2)	14 (29.8)	44 (25.1)	31 (52.5)	<0.001
At least moderate pain	12 (10.3)	5 (10.6)	31 (17.7)	10 (16.9)	0.273
≥1 ADL impairment	9 (7.8)	9 (19.1)	42 (24.0)	20 (33.9)	<0.001
≥1 IADL impairment	17 (14.7)	9 (19.1)	56 (32.0)	25 (42.4)	<0.001
MNA Total	12.8 ± 1.5	12.8 ± 1.7	12.8 ± 1.5	12.5 ± 1.6	0.444
Nutrition Status					0.113
Malnourished	0 (0.0)	1 (2.1)	0 (0.0)	1 (1.7)	
At Risk	22 (19.0)	4 (8.5)	28 (16.0)	14 (23.7)	
Normal	94 (81.0)	42 (89.4)	147 (84.0)	44 (74.6)	
Physical Performance					
Max gait speed, m/s	1.2 ± 0.2 ^a,b^	1.2 ± 0.2 ^c,d^	0.8 ± 0.2 ^a,c^	0.8 ± 0.2 ^b,d^	<0.001
Max handgrip strength, kg	23.4 ± 7.0 ^a^	23.6 ± 8.2 ^b^	21.5 ± 6.8 ^c^	18.2 ± 4.3 ^a,b,c^	<0.001
Low grip strength ^1^	52 (44.8)	18 (38.3)	104 (59.4)	39 (66.1)	0.002
SPPB, total	11.0 ± 1.3 ^a,b^	10.7 ± 1.5 ^c,d^	9.0 ± 2.2 ^a,c^	8.4 ± 2.3 ^b,d^	<0.001
5× STS time (s)	11.2 ± 2.9 ^a,b^	12.1 ± 3.0 ^c^	13.8 ± 4.9 ^a,d^	16.3 ± 7.1 ^b,c,d^	<0.001
Body Composition					
ASMI (kg/m^2^)	7.0 ± 2.4	7.0 ± 2.2	7.3 ± 2.7	6.5 ± 2.0	0.220
Body fat percentage (%)	31.1 ± 8.8 ^a^	31.9 ± 7.3	33.7 ± 9.4	36.2 ± 9.3 ^a^	0.007
Fat Mass Index	7.9 ± 3.2 ^a^	8.0 ± 2.7	9.0 ± 3.8	9.7 ± 4.1 ^a^	0.008
Fat Free Mass Index	16.8 ± 2.4	16.6 ± 1.9	16.8 ± 2.4	16.1 ± 1.7	0.205
Fat Mass to Fat Free Mass Ratio	0.5 ± 0.2 ^a^	0.5 ± 0.2	0.5 ± 0.2	0.6 ± 0.2 ^a^	0.003
Visceral fat area (cm^2^) *	94.1 ± 39.0	92.1 ± 32.9	105.9 ± 47.7	110.1 ± 51.2	0.095
Sarcopenia (AWGS) ^2^	1 (0.9)	1 (2.1)	36 (20.6)	17 (28.8)	<0.001

Values presented as *n* (%) or mean ± SD; * *n* = 223; ^abcd^ Values with a common superscript alphabet are significantly different. BMI: Body Mass Index; RAPA: Rapid Assessment of Physical Activity; MoCA: Montreal Cognitive Assessment; ADL: Activities of Daily Living; IADL: Instrumental Activities of Daily Living; MNA: Mini Nutritional Assessment; SPPB: Short Physical Performance Battery Test; STS: Sit-to-Stand; ASMI: Appendicular Skeletal Muscle Index; AWGS: Asian Working Group for Sarcopenia. ^1^ Adjusted for gender; ^2^ Based on Asian Working Group for Sarcopenia (AWGS) 2019’s definition.

**Table 2:** Univariate and multiple adjusted logistic regression for body composition, anthropometric measures and sarcopenia.

	Subjective Cognitive Decline		Slow Gait		Motoric Cognitive Risk Syndrome	
	Unadjusted	Adjusted ^#^	Unadjusted	Adjusted ^#^	Unadjusted	Adjusted ^#^
Waist Circumference (cm)	1.01 (1.03)	1.02 (0.96–1.04)	1.04 (1.01–1.06)	**1.04** **(1.01–1.07)**	1.02 (0.99–1.05)	1.03 (0.99–1.07)
Body Fat Percentage	1.01 (0.97–1.05)	1.00 (0.95–1.05)	**1.03** **(1.00–1.06)**	1.03 (0.99–1.07)	**1.07** **(1.03–1.11)**	**1.06** **(1.01–1.12)**
Fat Mass Index	1.02 (0.91–1.13)	0.99 (0.87–1.13)	1.10 (1.02–1.19)	1.10 (0.99–1.21)	**1.15** **(1.05–1.27)**	**1.16** **(1.02–1.30)**
Fat Free Mass Index	0.96 (0.82–1.12)	0.98 (0.81–1.19)	1.02 (0.91–1.13)	1.09 (0.95–1.26)	0.87 (0.74–1.01)	0.99 (0.81–1.21)
Fat Mass to Fat Free Mass ratio	1.28 (0.22–3.55)	0.78 (0.08–2.99)	**1.61** **(1.33–2.09)**	1.26 (0.68–1.83)	**1.44** **(1.04–4.41)**	**2.56** **(1.62–3.33)**
ASMI (kg/m^2^)	0.99 (0.85–1.16)	1.01 (0.83–1.23)	1.05 (0.95–1.17)	1.09 (0.95–1.25)	0.85 (0.69–1.06)	0.98 (0.78–1.24)
Sarcopenia ^	**2.35** **(2.00–5.60)**	2.83 (0.16–5.29)	**2.25** **(1.68–3.02)**	**1.90** **(1.14–2.40)**	**3.16** **(2.04–4.75)**	**2.62** **(1.46–3.17)**

Reference group: Healthy; Values presented as Odds Ratio (95% Confidence Interval); Bold indicates significance (*p* < 0.05). ^ As defined by Asian Working Group for Sarcopenia 2019. ASMI: Appendicular Skeletal Muscle Index. ^#^ adjusted for age, gender, ethnicity, education years, hypertension, hyperlipidemia, diabetes, polypharmacy, nutritional status and physical activity.

**Table 3:** Inflammatory biomarkers.

	Healthy	Subjective Cognitive Decline	Slow Gait	Motoric Cognitive Risk Syndrome	*p*-Value
	*n* = 36 (33.6%)	*n* = 22 (20.6%)	*n* = 33 (30.8%)	*n* = 16 (15.0%)	
GDF-15 (pg./mL)	715.6 ± 315.4	981.8 ± 506.4	1100.5 ± 565.0	1132.4 ± 602.5	0.076
Interleukin 6 (pg./mL)	2.7 ± 0.8	2.8 ± 0.8	3.1 ± 1.2	2.9 ± 0.7	0.404
Interleukin 10 (IL-10) (ng/mL)	2.4 ± 0.9	2.4 ± 0.9	2.3 ± 1.0	2.3 ± 1.0	0.968
Progranulin (ng/mL)	69.3 ± 11.8	68.8 ± 12.7	67.2 ± 14.1	67.5 ± 13.7	0.915
Tumor Necrosis-α (TNF-α) (pg/mL)	7.0 ± 1.7 ^a^	7.1 ± 1.4 ^b^	8.5 ± 2.8	9.5 ± 2.6 ^a,b^	**<0.001**
IL-10/TNF-α	396.9 ± 295.1 ^a^	306.2 ± 85.7	307.7 ± 184.6	222.6 ± 117.8 ^a^	**0.045**
Progranulin/TNF-α	10,124.4 ± 4018.4 ^a^	9563.2 ± 2461.7 ^b^	8744.7 ± 4006.7	6869.2 ± 2235.4 ^a,b^	**0.022**

^ab^ Values presented as mean ± SD; GDF-15: Growth Differentiation Factor-15; Bold indicates significance (*p* < 0.05).

**Table 4:** Univariate and multiple adjusted logistic regression for plasma biomarkers.

	Subjective Cognitive Decline			Slow Gait			Motor Cognitive Risk Syndrome		
	Model 1	Model 2	Model 3	Model 1	Model 2	Model 3	Model 1	Model 2	Model 3
GDF-15	1.00 (0.99–1.01)	1.00 (0.98–1.02)	1.00 (0.97–1.03)	1.01 (1.00–1.02)	1.01 (0.99–1.02)	1.01 (0.99–1.03)	**1.01** **(1.00–1.02)**	1.01 (0.99–1.03)	1.02 (0.99–1.04)
IL-6	1.23 (0.80–1.87)	0.88 (0.36–2.10)	0.69 (0.22–2.16)	1.26 (0.86–1.85)	0.58 (0.23–1.42)	0.19 (0.04–1.08)	1.19 (0.74–1.90)	0.79 (0.33–1.91)	0.57 (0.11–2.87)
IL-10	1.02 (0.95–1.09)	1.06 (0.93–1.20)	1.03 (0.89–1.18)	1.02 (0.96–1.09)	1.07 (0.94–1.21)	1.04 (0.91–1.20)	0.87 (0.53–1.44)	0.68 (0.27–1.75)	**0.22** **(0.05–0.98)**
PRGN	0.99 (0.96–1.04)	0.96 (0.88–1.04)	0.96 (0.88–1.05)	0.99 (0.95–1.03)	0.96 (0.90–1.02)	0.96 (0.89–1.04)	0.99 (0.94–1.04)	0.97 (0.90–1.04)	0.99 (0.91–1.09)
TNF-α	0.97 (0.80–1.18)	0.72 (0.41–1.26)	0.87 (0.45–1.69)	1.09 (0.94–1.28)	0.99 (0.73–1.35)	1.23 (0.79–1.93)	**1.23** **(1.04–1.46)**	**1.37** **(1.01–1.87)**	1.55 (0.99–2.43)
IL-10/TNF-α	0.98 (0.95–1.01)	0.99 (0.98–1.08)	0.99 (0.97–1.01)	0.99 (0.95–1.01)	0.99 (0.98–1.04)	0.99 (0.98–1.04)	**0.98** **(0.97–0.99)**	**0.98** **(0.97–0.99)**	**0.98** **(0.97–0.99)**
PRGN/ TNF-α	1.00 (1.00–1.00)	1.00 (1.00–1.00)	1.00 (1.00–1.00)	1.00 (1.00–1.00)	1.00 (1.00–1.00)	1.00 (1.00–1.00)	**0.98** **(0.92–0.99)**	**0.98** **(0.96–0.99)**	1.00 (0.99–1.00)

Reference group: Healthy; Values presented as Odds Ratio (95% Confidence Interval); Bold indicates significance (*p* < 0.05); GDF-15: Growth Differentiation Factor-15; IL6, Interleukin 6; IL-10: Interleukin 10; TNF-α: Tumor Necrosis Factor Alpha; PRGN: Progranulin. Model 1: Unadjusted. Model 2: Adjusted for age, gender, ethnicity, education years, hypertension, hyperlipidemia, diabetes, polypharmacy, nutritional status, physical activity and sarcopenia. Model 3: Adjusted for Model 2 and body fat percentage.

## Data Availability

The data presented in the current study are available from the corresponding author on reasonable request.
